# Complicated Appendicitis Among Adults With and Without Disabilities: A Cross-Sectional Nationwide Study in South Korea

**DOI:** 10.3389/fpubh.2022.813608

**Published:** 2022-04-04

**Authors:** Kyoung Eun Yeob, So Young Kim, Jong Eun Park, Jong Hyock Park

**Affiliations:** ^1^Institute of Health and Science Convergence, Chungbuk National University, Cheongju, South Korea; ^2^Department of Public Health and Preventive Medicine, Chungbuk National University Hospital, Cheongju, South Korea; ^3^Harvard T.H. Chan School of Public Health, Harvard University, Boston, MA, United States

**Keywords:** disability, complicated appendicitis, disparity, healthcare access, Korea

## Abstract

**Objective:**

Appendicitis is usually diagnosed based on a reliable set of signs and symptoms, and can be effectively treated with surgery, with low morbidity and mortality rates. However, appendicitis is often overlooked in vulnerable populations, including people with disabilities. This study compared 10-year trends of complicated appendicitis between South Koreans with a disability, according to disability severity and type, and those without disabilities

**Methods:**

To identify cases of appendicitis, we used the DRG codes in the National Health Information Database of South Korea. Patients with appendicitis were classified in terms of severity based on the DRG codes. Age-standardized incidence rates were calculated for each year during 2008–2017 according to the presence, type, and severity of the disability. Factors associated with complicated appendicitis were examined by multivariate logistic regression using the most recent data (i.e., 2016–2017).

**Results:**

The incidence of complicated appendicitis was higher in people with disabilities, especially those with severe disabilities (26.9 vs. 11.6%). This difference was particularly marked when considering those with a severe disability (aOR = 1.868, 95% CI:1.511–2.309), internal organ problems (aOR = 10.000, 95% CI:5.365–18.638) or a mental disability (aOR = 2.779, 95% CI:1.563–4.939).

**Conclusions:**

The incidence of complicated appendicitis was higher in people with disability than in those without disability in all years. There was a substantial difference in the incidence of complicated appendicitis between the severe disability and non-disabled groups. Among the various disability types, the incidence of complicated appendicitis was highest for major internal organ problems, followed by intellectual or psychological disabilities. Our findings may be explained by barriers to healthcare access among people with disabilities, particularly those with a severe disability, internal organ problem, or mental disability.

## Introduction

Appendectomy is one of the most common operations performed worldwide, including in South Korea (1). Appendicitis is typically diagnosed according to a reliable set of signs and symptoms, and can be effectively treated with surgery, with low morbidity and mortality rates. However, appendicitis is often overlooked in vulnerable populations, including people with disabilities (2).

A delay in the diagnosis and treatment of some conditions can result in serious adverse outcomes; delayed diagnosis and treatment of appendicitis can result in appendiceal rupture, peritonitis, and death (3). The risk of rupture is negligible within the first 24 h; however, the rupture rate reaches 6% 36 h after the onset of symptoms (4, 5).

It has been suggested that rupture rate of appendicitis could serve as an indicator of access to healthcare (6). In the US, racial/ethnic differences in the rate of poor outcomes of acute appendicitis, such as perforation of the appendix or complicated disease, have been reported (6). Appendectomy is the most common intra-abdominal surgical procedure (6), and has no known behavioral or social risk factors and only one treatment option (appendectomy). Appendectomy should be performed urgently, regardless of the time of day.

Timely and appropriate healthcare plays a key role in wellness, illness prevention, and optimal recovery when illness occurs (7). Several studies have reported healthcare disparities between people with and without disabilities; the former group are more likely to experience a delay in healthcare. These disparities contribute to differences in outcomes, such as mortality (8–11). Such disparities have been reported in studies of women with disabilities being screened for cervical cancer or undergoing dental examinations, as well in studies on the diagnosis and treatment of cancer (12–14).

Although numerous epidemiological studies on complicated appendicitis have been conducted, most focused on children or older adults; relatively few focused on people with disabilities, especially with a long observation period. Previous studies were mainly concerned with the incidence of complicated appendicitis (15–17), differences in the incidence of complicated appendicitis between patients with and without disabilities, and factors affecting complicated appendicitis in people with disabilities. Our target population was people with disabilities diagnosed with complicated appendicitis; the comparison group was patients with complicated appendicitis without disabilities and the outcome variable was the incidence of complicated appendicitis. To explore potential differences in the incidence of complicated appendicitis between patients with and without disabilities, we conducted a cross-sectional study.

## Materials and Methods

### Data Source and Study Subjects

**Figure 1:**
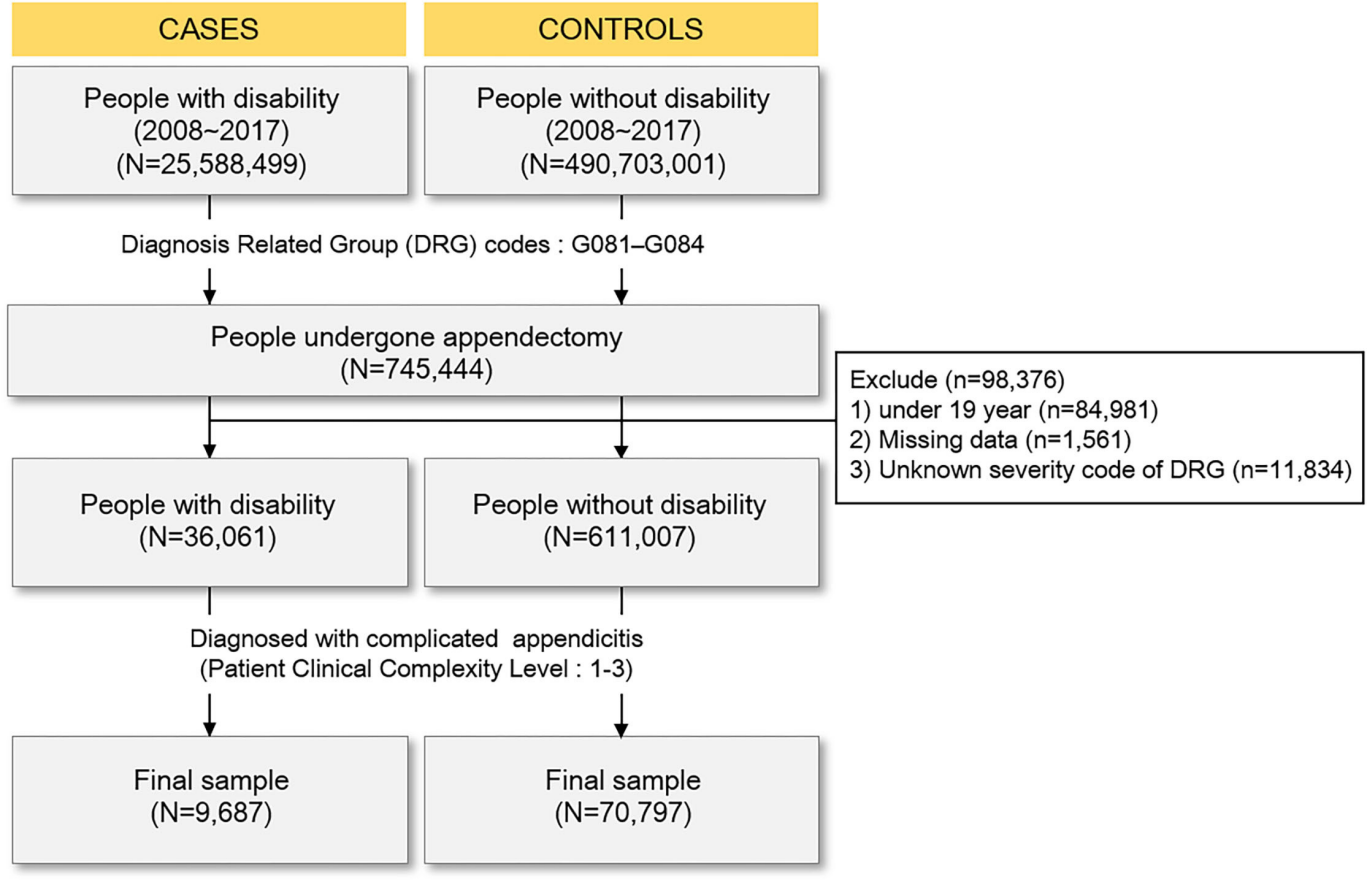
Flow chart.

This study used information from the National Health Insurance Service (NHIS) database of the National Health Insurance Sharing Service. The Korean NHIS covers 97% of the Korean population; only Medical Aid beneficiaries in the lowest income bracket are not covered. The NHIS contains information about age, sex, residential area, monthly insurance contributions (a proxy for income status), disability type and severity, and vital statistics. The NHIS claims database enables easy retrieval and analysis of population-based epidemiological data. For this study, population-based medical data for patients of all ages with appendicitis were retrospectively extracted from the NHI claims database from January 2008 to December 2017. We collected information on disability severity and type from Using a disability registry. The database covered 93.8% of the total disabled population as of 2011 (18). Using Korean personal identification numbers, disability severity and type were linked with variables selected from the NHIS claims database. We excluded patients aged <19 years at the time of diagnosis (*n* = 84,981), as well as those who had missing data (*n* = 1,561) or an appendectomy of unknown severity (i.e., missing codes; *n* = 11,834) (Figure 1). During the study period, 6,47,068 patients were screened for eligibility. Finally, the study sample included 9,687 patients with disabilities upon their complicated appendicitis diagnosis and 70,797 patients without disabilities upon the complicated appendicitis diagnosis during a 10-year period from 2008 to 2017.

### Definition of Complicated Appendicitis, and Other Variables

The primary study outcome of the rate of complicated appendicitis. Patients with complicated appendicitis were classified based on KDRG codes according to severity. Cases of appendicitis treated with appendectomy were identified using diagnosis-related group (DRG) codes G081–G084. The DRG-based payment system in South Korea is a case-based prospective payment system under which payments are made according to physician-determined diagnoses (19). The Korean Diagnosis Related Group (KDRG) comprises eight surgical diseases and procedures (appendectomy, tonsillectomy, hernia, cesarean section, hysterectomy, eutocia, cataract, and hemorrhoids). Each code was subdivided according to the severity of the complications/comorbidities [Patient Clinical Complexity Level (PCCL): 0 = No Clinical Complexity (CC); 1 = Minor CC; 2 = Moderate CC; 3 = Severe CC]. Severity codes 1–3 were defined as complicated appendicitis. Other variables collected from the NHIS included age, sex, insurance premium, residential area, and comorbidities. Insurance premiums for each household were calculated based on income, property, and automobile taxes (20). Residential area was classified as metropolitan, urban, or rural based on the ZIP code. The subjects were classified into four categories based on the Charlson Comorbidity Index (CCI): 0, 1–2, 3–4, and ≥5 (the most severe) (21).

### Statistical Analysis

Descriptive statistics were generated on disability status (present or absent) and the type and severity of disability. Age-standardized incidence rates were calculated using 2005 Korean census data as the reference. To examine the association between disability and the incidence of complicated appendicitis, we conducted a series of multivariate logistic regression analyses adjusted for age, income level, place of residence, smoking status, the CCI score, diabetes, hypertension, chronic obstructive pulmonary disease, coronary artery disease, obesity, and malignancies (16, 22, 23) using the most recent dataset available (2016–17). These variables were all treated as categorical in the analyses (e.g., “presence” or “absence” of diabetes, hypertension, chronic obstructive pulmonary disease, etc.,). For missing data, we applied the listwise deletion method; although this can lead to the omission of many cases, which affects the statistical power of the tests conducted (24, 25), if the percentage of missing data is very small or the sample is sufficiently large, the power should still be sufficient to detect meaningful effects. This study analyzed the national NHIS claims database, which includes valid and accurate information, especially on socioeconomic status and healthcare utilization, and has very little missing data (26). Given the large size of our sample, which included most of the general population with and without disabilities, listwise deletion did not adversely affect the statistical power. All analyses were performed using SAS software (version 9.3; SAS Institute, Cary, NC, USA), and a *p*-value < 0.05 was considered significant. This study was approved by the Institutional Review Board of Chungbuk National University (CBNU-202010-HRHR-0717).

## Results

### Study Participants

Of the non-disabled patients, 50.9% (*n* = 3,10,833) were male and 49.1% (*n* = 3,00,174) were female, compared to 61.0% (*n* = 21,990) and 39.0% (*n* = 14,071) among the patients with a disability, respectively (<0.0001). Patients with appendicitis and a disability were slightly older than the control subjects (males: 56.8 vs. 42.5 years, females: 62.0 vs. 43.8 years, <0.0001). The economic status of the cohort with disabilities was lower than that of those without a disability. The proportion of patients with medical aid was higher in the disabled group, but the number in the fifth (highest) quartile was larger in the non-disabled group (<0.0001). People with versus without disabilities had more comorbidities and a higher mean CCI score (males: 2.3 vs. 1.0, females: 2.4 vs. 1.1, <0.0001), and were more likely to live in a rural area (<0.0001). In total, 12.2 and 11.0% of patients without a disability (<0.0001), and 27.3 and 26.2% of those with a disability (*p* = 0.0209), had been diagnosed with complicated appendicitis (Table 1).

**Table 1:** Baseline characteristics of study population in South Korea during 2008–2017 according to disability status and sex.

			**People without disability**				***P*-value**	**People with disability**				***P*-value**
	**Total**		**Male**		**Female**			**Male**		**Female**		
	***N***	**%**	***n***	**%**	***N***	**%**		***n***	**%**	***n***	**%**	
Total		647,068		310,833 (50.9)		300,174 (49.1)		21,990 (61.0)		14,071 (39.0)		
**Age**												
Mean ± SD	44.0 ± 16.3		42.5 ± 15.4		43.8 ± 16.5			56.8 ± 15.5		62.0 ± 15.6		
95% confidence intervals	43.960–44.040		42.446–42.554		43.741–43.859			56.595–57.005		61.742–62.258		
20–29	144,369	22.3	72,937	23.5	69,805	23.3	<0.0001	1,121	5.1	506	3.6	<0.0001
30–39	151,022	23.3	79,320	25.5	68,608	22.9		2,215	10.1	879	6.2	
40–49	126,246	19.5	63,621	20.5	57,308	19.1		3,676	16.7	1,641	11.7	
50+	225,431	34.8	94,955	30.5	104,453	34.8		14,978	68.1	11,045	78.5	
**Income level**												
Medical aid and First quartile (lowest)	109,065	16.9	41,723	13.4	55,712	18.6	<0.0001	6,845	31.1	4,785	34.0	<0.0001
Second quartile	104,214	16.1	46,491	15.0	53,398	17.8		2,796	12.7	1,529	10.9	
Third quartile	123,176	19.0	62,511	20.1	55,537	18.5		3,344	15.2	1,784	12.7	
Fourth quartile	139,804	21.6	71,502	23.0	61,700	20.6		4,171	19.0	2,431	17.3	
Fifth quartile (highest)	157,163	24.3	81,356	26.2	67,921	22.6		4,532	20.6	3,354	23.8	
Unknown	13,646	2.1	7,250	2.3	5,906	2.0		302	1.4	188	1.3	
**Residence**												
Metropolitan	396,448	61.3	190,916	61.4	186,941	62.3	<0.0001	11,412	51.9	7,179	51.0	<0.0001
City	182,953	28.3	88,408	28.4	83,107	27.7		7,069	32.1	4,369	31.0	
Rural	65,386	10.1	30,141	9.7	29,277	9.8		3,466	15.8	2,502	17.8	
Unknown	2,281	0.4	1,368	0.4	849	0.3		43	0.2	21	0.1	
**Charlson comorbidity index**												
Mean ± SD	1.1 ± 1.7		1.0 ± 1.6		1.1 ± 1.5			2.3 ± 2.5		2.4 ± 2.3		
95% confidence intervals	1.096–1.104		1.094–1.106		1.095–1.105			2.267–2.333		2.362–2.438		
0	314,808	48.7	165,127	53.1	140,737	46.9	<0.0001	5,991	27.2	2,953	21.0	<0.0001
1~2	242,931	37.5	107,656	34.6	121,709	40.5		8,024	36.5	5,542	39.4	
3~4	60,023	9.3	25,489	8.2	26,981	9.0		4,359	19.8	3,194	22.7	
≥5	29,306	4.5	12,561	4.0	10,747	3.6		3,616	16.4	2,382	16.9	
Complicated appendicitis												
No	566,584	87.6	272,957	87.8	267,253	89.0	<0.0001	15,988	72.7	10,386	73.8	0.0209
Yes	80,484	12.4	37,876	12.2	32,921	11.0		6,002	27.3	3,685	26.2	

#### Incidence of Complicated Appendicitis According to the Presence and Absence of a Disability for 2008–2017

**Figure 2:**
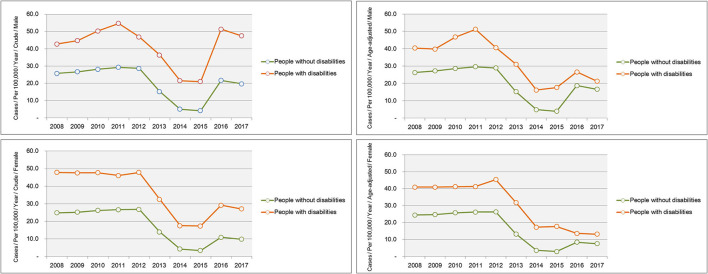
Trends in the incidence of complicated appendicitis according to the presence and absence of a disability for 2008–2017.

Trends in the crude and age-adjusted incidence rates of complicated appendicitis per 1,00,000 population according to the presence or absence of a disability are shown in Figure 2. The age-adjusted incidence of complicated appendicitis according to the presence or absence of a disability decreased gradually from 2008 to 2017, but the incidence was higher in disabled than non-disabled males and females in all years. The age-adjusted incidence rates of complicated appendicitis in males and females were 21.2 and 13.2 per 1,00,000 population (2017) in people with disabilities, and 16.6 and 7.5 (2017) per 1,00,000 population in people without disabilities, respectively (see Supplementary Table 1).

#### Incidence of Complicated Appendicitis According to the Severity of Disability for 2008–2017

**Figure 3:**
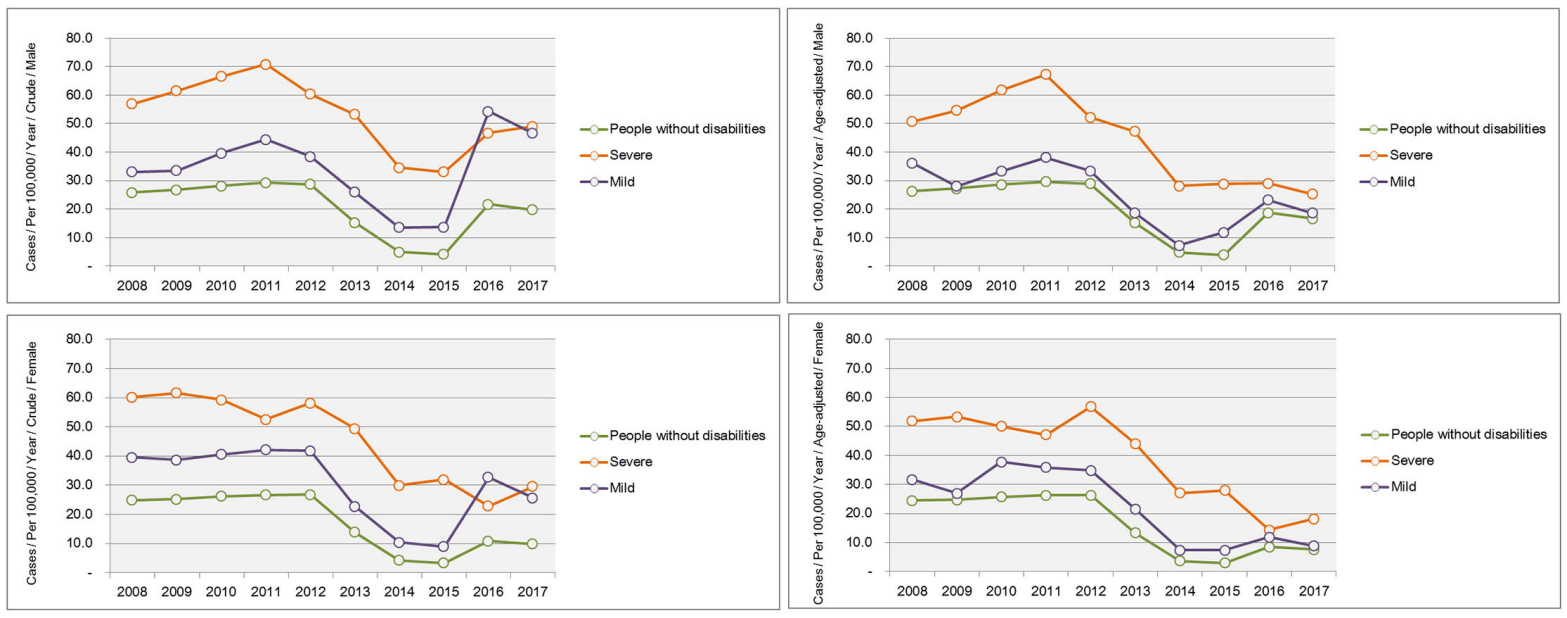
Trends in the incidence of complicated appendicitis according to the severity of a disability for 2008–2017.

Trends in the crude and age-adjusted incidence rates of complicated appendicitis per 1,00,000 population according to the severity of disability are shown in Figure 3. The greatest group difference in the incidence of complicated appendicitis was seen between the severe disability and non-disabled groups. In particular, in 2017 the age-adjusted incidence for severely disabled males was 1.5 times higher than that of non-disabled people (age-adjusted incidence per 1,00,000 population: 25.2 vs. 16.6) and severely disabled females was 2.4 times higher than that of non-disabled people (age-adjusted incidence per 1,00,000 population: 18.2 vs. 7.35) (see Supplementary Table 2).

#### Incidence of Complicated Appendicitis According to Type of Disability for 2008–2017

**Figure 4:**
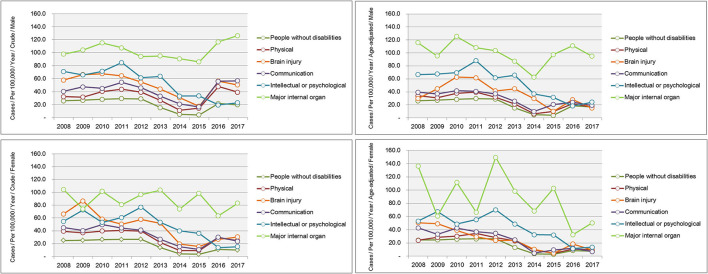
Trends in the incidence of complicated appendicitis according to the type of a disability for 2008–2017.

Trends in the crude and age-adjusted incidence rates of complicated appendicitis per 1,00,000 population according to the type of disability are shown in Figure 4. Among the various disability types, the highest incidence of age-adjusted complicated appendicitis was observed for major internal organ problems (e.g., 94.8 and 50.4 males and females per 1,00,000 population in 2017, respectively), followed by intellectual or psychological disabilities (e.g., 24.1 and 13.7 males and females per 1,00,000 population in 2017, respectively) in every year (see Supplementary Table 3).

#### Factors Associated With Complicated Appendicitis During 2016–2017

Males and females with disabilities had a higher incidence of complicated appendicitis than those without a disability [adjusted odds ratio (aORs) = 1.204 and 1.389 for males and females, respectively]. This difference was greater in those with a severe disability (aORs = 1.792 and 1.894 for males and females, respectively). The risk was high for males in the internal organ problems [aOR = 5.581, 95% confidence interval (CI): 3.047–10.222] and intellectual/psychological disabilities (aOR = 2.790, 95% CI: 1.281–6.073) groups, particularly in those with severe internal organ problems (aOR = 10.857, 95% CI: 5.247–22.465) and intellectual/psychological disorders (aOR = 2.818, 95% CI: 1.295–6.136). Similarly, the risk of complicated appendicitis was particularly high in females with severe internal organ problems (aOR = 7.660, 95% CI: 2.297–25.545) (Table 2).

**Table 2:** Factors associated with complicated appendicitis during 2016–2017.

	**Male: OR (95% CI)**		**Female: OR (95% CI)**	
	Model 1^*^	Model 2^**^	Model 1^*^	Model 2^**^
**Disability**				
Yes (vs. no)	3.149 (2.951–3.360)	1.204 (1.059–1.369)	3.972 (3.609–4.370)	1.389 (1.135–1.701)
**By disability severity**				
Severe (vs. no)	4.377 (3.928–4.879)	1.792 (1.394–2.304)	4.905 (4.166–5.775)	1.894 (1.251–2.868)
Mild (vs. no)	2.713 (2.509–2.933)	1.068 (0.924–1.234)	3.625 (3.234–4.064)	1.278 (1.020–1.602)
**By disability type**				
Physical (vs. no)	2.451 (2.241–2.681)	1.018 (0.863–1.200)	3.414 (2.997–3.889)	1.306 (1.019–1.675)
Brain injury (vs. no)	4.891 (3.985–6.003)	2.102 (1.297–3.406)	4.467 (3.339–5.976)	1.109 (0.522–2.359)
Communication (vs. no)	2.945 (2.605–3.329)	1.133 (0.904–1.419)	3.541 (2.913–4.303)	1.184 (0.774–1.812)
Intellectual or psychological (vs. no)	2.579 (1.937–3.435)	2.790 (1.281–6.073)	3.037 (2.072–4.450)	2.413 (0.984–5.920)
Major internal organ (vs. no)	19.581 (15.230–25.174)	5.581 (3.047–10.222)	25.247 (17.544–36.332)	6.901 (2.731–17.433)
**By disability type and severe**				
**Physical**				
Severe (vs. no)	2.867 (2.340–3.514)	1.300 (0.873–1.936)	3.404 (2.340–4.952)	1.116 (0.514–2.421)
Mild (vs. no)	2.370 (2.147–2.616)	0.973 (0.814–1.164)	3.415 (2.976–3.920)	1.329 (1.024–1.725)
**Brain injury**				
Severe (vs. no)	4.728 (3.555–6.288)	1.777 (0.85–3.715)	4.330 (2.949–6.359)	1.914 (0.696–5.266)
Mild (vs. no)	5.070 (3.783–6.795)	2.390 (1.269–4.501)	4.660 (2.993–7.257)	0.570 (0.173–1.884)
**Communication**				
Severe (vs. no)	3.014 (2.361–3.847)	1.132 (0.713–1.797)	3.461 (2.403–4.986)	1.598 (0.692–3.691)
Mild (vs. no)	2.922 (2.539–3.363)	1.135 (0.879–1.467)	3.572 (2.841–4.491)	1.074 (0.658–1.755)
**Intellectual or psychological**				
Severe (vs. no)	2.593 (1.947–3.453)	2.818 (1.295–6.136)	3.037 (2.072–4.450)	2.407 (0.981–5.908)
Mild (vs. no)				
**Major internal organ**				
Severe (vs. no)	27.109 (19.863–36.999)	10.857 (5.247–22.465)	32.041 (20.639–49.743)	7.660 (2.297–25.545)
Mild (vs. no)	8.430 (5.291–13.432)	1.303 (0.474–3.582)	13.665 (6.899–27.064)	5.947 (1.444–24.495)

* *Crude*.

** *Adjusted for age, Income level, area of residence, smoking, obesity, CCI, diabetes, hypertension, COPD, coronary artery disease, and any malignancy*.

## Discussion

This is the first study to comprehensively analyze potential disparities in the incidence of complicated appendicitis according to disability status. The strengths of this study included the large number of participants, who were representative of the entire population of South Korea, and the accurate disability diagnoses.

The incidence of complicated appendicitis has been continuously decreasing in both disabled and non-disabled patient groups since 2011. The incidence fell particularly sharply from 2014 to 2015, as also reported in previous studies based on KDRG codes. According to Shin, the ratio of the 1–3 to 0 severity classes decreased 0.49-fold during 2014–2015 among health insurance and medical aid patients. This obviously suggests a decrease in the proportion of high-severity patients, but may also reflect more accurate claims and KDRG code data in association with the introduction of a new payment system in 2013 (27). Further study on this topic is necessary.

In this study, the incidence rates of complicated appendicitis were higher in disabled people compared to those without disabilities. The main drivers of complicated appendicitis are considered to be delayed diagnosis and treatment. Although we could not establish the reasons for late diagnosis and treatment in our disabled group, barriers to healthcare have been suggested in previous studies, including access to facilities, equipment, and transportation (7, 28, 29). Accessing medical facilities is more difficult for disabled than non-disabled people due to physical and transportation problems; this leads to late diagnosis or treatment of appendicitis, which in turn increases the likelihood of complicated appendicitis developing. Efforts are needed to resolve this problem, such as increasing the number of accessible facilities and availability of medical equipment, and providing support for transportation. People with disabilities may also face financial barriers due to the cost of diagnosis and treatment. According to a previous study of people with disabilities in Australia, one in four (24%) delayed going, or did not go, to a hospital; one in five (19%) delayed or did not see a general practitioner, and one in four (27%) did not see a medical specialist because of the cost (30). Thus, financial barriers may lead to delayed diagnosis or treatment of complicated appendicitis. In addition, limitations of healthcare providers themselves (e.g., poor knowledge, negative attitudes, lack of time, and failure to prioritize disabled people in the face of multiple demands), and patient factors (e.g., lack of knowledge and access to the usual source of care), are associated with complicated appendicitis (28, 31, 32). Thus, policies aimed at improving attitudes and access to the usual source of care are needed to decrease the incidence of complicated appendicitis among the disabled.

In our study, the incidence of complicated appendicitis was highest among patients with internal organ problems; these patients have more comorbidities than those with other types of disabilities (14, 33, 34). The CCI score predicts mortality due to acute appendicitis. A CCI score > 5 (OR = 52.45, *p* < 0.05) was shown to be an independent predictor of mortality due to acute appendicitis (22). Another study of perforated appendicitis in Asians reported that the risk of perforation was higher in patients with one or more comorbidities (15). Therefore, the presence of serious comorbidities is associated with a worse prognosis even for a relatively benign disease, and even in the absence of complications. Patients with a disability due to renal failure have a high incidence of complicated appendicitis. Patients on long-term dialysis undergoing non-emergent procedures are at high risk for complications; an operative mortality rate of 13% has been reported (34). Patients with a disability due to renal failure lack adequate kidney function, so must rely on dialysis to regulate fluid and electrolyte balance, as well as the metabolism of drugs and toxins (35). These problems make postoperative and intraoperative monitoring of a disability due to renal failure challenging, and limit the pharmacological options for surgeons and anesthesiologists (35). As a result, patients with a disability due to renal failure are at increased risk of morbidity and mortality in association with operative procedures.

Our patients with intellectual or psychological disabilities had a high incidence of complicated appendicitis. These results are consistent with previous studies showing that patients with severe intellectual disabilities have more comorbidities than the general population (36), and are susceptible to delayed diagnosis, adverse surgical outcomes, impaired communication, pain and adverse drug reactions (37). Lin reported that surgical patients with an intellectual disability are at higher risk of many complications compared to the general population, including acute renal failure (OR = 3.81, 95% CI: 2.28–6.37), pneumonia (OR = 2.01, 95% CI: 1.61–2.49), postoperative bleeding (OR = 1.35, 95% CI: 1.09–1.68), and septicemia (OR = 2.43, 95% CI: 1.85–3.21) (38). These findings show that strategies are needed to reduce postoperative adverse outcomes in this population.

Our study had several limitations. First, it was retrospective, so we were unable to collect data on all factors that may have affected the outcomes, such as clinical data (e.g., fever, white blood cell count, type of appendicitis, and type of surgery). We believe that adjusting for other covariates in the multivariate model would be sufficient to address the confounding effect of differences in patient characteristics between people with and without disabilities, in terms of risk factor identification. Matching the subjects could prevent such confounding. Second, we could not ascertain why some patients were more at risk of complicated appendicitis, where potential reasons include patient or family refusal to undergo treatment, economic/transportation problems, or clinical decision-making. Further studies using other research methods and statistical analyses (e.g., calculation of absolute and relative risk), including patient surveys and interviews, are required to precisely determine how these factors affect the incidence of complicated appendicitis. Third, although we included subjects with the DRG code for appendectomy, whether they were actually diagnosed with appendicitis was unclear, and patients who did not undergo surgery (e.g., those treated with antibiotics only) were not included (although the number of such patients was small). However, the main treatment for appendicitis is appendectomy; previous studies reported that more than 96% of appendicitis patients underwent this surgery (39). Therefore, most of the patients in this study likely had appendicitis.

## Conclusion

Our findings indicate disparities in access to healthcare between non-disabled and disabled populations, particularly for those with severe or mental disabilities, or internal organ problems. Although the disparity might in part be due to clinical decision-making, unequal access to healthcare for people with disabilities is unjustifiable. Public health policies should focus on people with disabilities to reduce disparities in health outcomes. Healthcare professionals, as well as people with disabilities and their families, should be educated to improve attitudes, and regarding the need for equal access to diagnosis and treatment.

## Data Availability Statement

The original contributions presented in the study are included in the article/Supplementary Materials, further inquiries can be directed to the corresponding author.

## Author Contributions

KEY, SYK, JEP, and JHP: conceptualization. JEP and JHP: data curation. JEP: formal analysis. KEY, SYK, and JHP: funding acquisition. JEP and KEY: investigation and methodology. SYK: project administration. JHP: resources. KEY and SYK: software, supervision, validation, visualization, roles/writing—original draft, and writing—review and editing. All authors contributed to the article and approved the submitted version.

## Funding

This work was supported by National Research Foundation of Korea (NRF) grants funded by the Ministry of Education (Nos. 2020R1I1A1A01054268 and 2019R1D1A3A03103862) and by the Korean Government (MSIT) (No. 2019R1A2C1087507).

## Conflict of Interest

The authors declare that the research was conducted in the absence of any commercial or financial relationships that could be construed as a potential conflict of interest.

## Publisher's Note

All claims expressed in this article are solely those of the authors and do not necessarily represent those of their affiliated organizations, or those of the publisher, the editors and the reviewers. Any product that may be evaluated in this article, or claim that may be made by its manufacturer, is not guaranteed or endorsed by the publisher.
