# Supplementary material for: Complicated Appendicitis Among Adults With and Without Disabilities: A Cross-Sectional Nationwide Study in South Korea
Source: Front Public Health. 2022 Apr 4;10:813608. doi: 10.3389/fpubh.2022.813608 (PMC9013817; doi:10.3389/fpubh.2022.813608)
Supplement: Supplementary file 1 [file Table_1.DOCX]

**Supplementary Table 1. Incidence rate of complicated appendicitis according to presence or absence of disability**

| **Year** | **2008** | **2009** | **2010** | **2011** | **2012** | **2013** | **2014** | **2015** | **2016** | **2017** |
| --- | --- | --- | --- | --- | --- | --- | --- | --- | --- | --- |
| Male |  |  |  |  |  |  |  |  |  |  |
| By disability |  |  |  |  |  |  |  |  |  |  |
| People without disability |  |  |  |  |  |  |  |  |  |  |
| Total | 17,672,422 | 17,827,464 | 18,040,675 | 18,319,643 | 18,595,507 | 18,845,674 | 19,143,089 | 19,427,929 | 19,662,987 | 19,857,936 |
| Patients of complicated appendicitis | 4,554 | 4,760 | 5,079 | 5,361 | 5,331 | 2,871 | 948 | 804 | 4,258 | 3,910 |
| Incidence rate (crude) | 25.8 | 26.7 | 28.2 | 29.3 | 28.7 | 15.2 | 5.0 | 4.1 | 21.7 | 19.7 |
| Incidence rate (age-adjusted) | 26.2 | 27.2 | 28.6 | 29.6 | 28.9 | 15.3 | 4.9 | 3.9 | 18.7 | 16.6 |
| People with disability |  |  |  |  |  |  |  |  |  |  |
| Total | 1,345,419 | 1,415,266 | 1,449,062 | 1,457,495 | 1,453,833 | 1,446,149 | 1,440,146 | 1,441,355 | 1,451,617 | 1,465,420 |
| Patients of complicated appendicitis | 576 | 634 | 730 | 798 | 682 | 527 | 310 | 303 | 746 | 696 |
| Incidence rate (crude) | 42.8 | 44.8 | 50.4 | 54.8 | 46.9 | 36.4 | 21.5 | 21.0 | 51.4 | 47.5 |
| Incidence rate (age-adjusted) | 40.4 | 39.8 | 46.7 | 51.2 | 40.6 | 31.0 | 16.2 | 17.7 | 26.6 | 21.2 |
| Female |  |  |  |  |  |  |  |  |  |  |
| By disability |  |  |  |  |  |  |  |  |  |  |
| People without disability |  |  |  |  |  |  |  |  |  |  |
| Total | 18,473,885 | 18,612,288 | 18,791,617 | 19,046,602 | 19,306,729 | 19,552,198 | 19,820,136 | 20,076,900 | 20,310,612 | 20,516,731 |
| Patients of complicated appendicitis | 4,595 | 4,689 | 4,925 | 5,071 | 5,172 | 2,722 | 839 | 679 | 2,215 | 2,014 |
| Incidence rate (crude) | 24.9 | 25.2 | 26.2 | 26.6 | 26.8 | 13.9 | 4.2 | 3.4 | 10.9 | 9.8 |
| Incidence rate (age-adjusted) | 24.4 | 24.7 | 25.7 | 26.2 | 26.3 | 13.2 | 3.6 | 2.9 | 8.4 | 7.5 |
| People with disability |  |  |  |  |  |  |  |  |  |  |
| Total | 898,242 | 991,966 | 1,037,101 | 1,049,002 | 1,049,532 | 1,046,467 | 1,044,426 | 1,045,534 | 1,056,301 | 1,071,485 |
| Patients of complicated appendicitis | 430 | 472 | 495 | 483 | 502 | 341 | 183 | 181 | 308 | 290 |
| Incidence rate (crude) | 47.9 | 47.6 | 47.7 | 46.0 | 47.8 | 32.6 | 17.5 | 17.3 | 29.2 | 27.1 |
| Incidence rate (age-adjusted) | 41.0 | 40.9 | 41.2 | 41.3 | 45.4 | 31.8 | 17.3 | 17.7 | 13.5 | 13.2 |

**Supplementary Table 2. Incidence rate of complicated appendicitis according to severity of disability**

| **Year** | **2008** | **2009** | **2010** | **2011** | **2012** | **2013** | **2014** | **2015** | **2016** | **2017** |
| --- | --- | --- | --- | --- | --- | --- | --- | --- | --- | --- |
| Male |  |  |  |  |  |  |  |  |  |  |
| By severity |  |  |  |  |  |  |  |  |  |  |
| People without disability |  |  |  |  |  |  |  |  |  |  |
| Total | 17,672,422 | 17,827,464 | 18,040,675 | 18,319,643 | 18,595,507 | 18,845,674 | 19,143,089 | 19,427,929 | 19,662,987 | 19,857,936 |
| Patients of complicated appendicitis | 4,554 | 4,760 | 5,079 | 5,361 | 5,331 | 2,871 | 948 | 804 | 4,258 | 3,910 |
| Incidence rate (crude) | 25.8 | 26.7 | 28.2 | 29.3 | 28.7 | 15.2 | 5.0 | 4.1 | 21.7 | 19.7 |
| Incidence rate (age-adjusted) | 26.2 | 27.2 | 28.6 | 29.6 | 28.9 | 15.3 | 4.9 | 3.9 | 18.7 | 16.6 |
| Severe (Grade 1-3) |  |  |  |  |  |  |  |  |  |  |
| Total | 549,543 | 572,513 | 578,482 | 572,316 | 563,684 | 555,870 | 550,834 | 548,070 | 548,062 | 548,774 |
| Patients of complicated appendicitis | 313 | 352 | 385 | 405 | 340 | 296 | 190 | 181 | 256 | 269 |
| Incidence rate (crude) | 57.0 | 61.5 | 66.6 | 70.8 | 60.3 | 53.2 | 34.5 | 33.0 | 46.7 | 49.0 |
| Incidence rate (age-adjusted) | 50.7 | 54.6 | 61.7 | 67.2 | 52.2 | 47.2 | 28.2 | 28.8 | 29.0 | 25.2 |
| Mild (Grade 4-6) |  |  |  |  |  |  |  |  |  |  |
| Total | 795,876 | 842,753 | 870,580 | 885,179 | 890,149 | 890,279 | 889,312 | 893,285 | 903,555 | 916,646 |
| Patients of complicated appendicitis | 263 | 282 | 345 | 393 | 342 | 231 | 120 | 122 | 490 | 427 |
| Incidence rate (crude) | 33.0 | 33.5 | 39.6 | 44.4 | 38.4 | 25.9 | 13.5 | 13.7 | 54.2 | 46.6 |
| Incidence rate (age-adjusted) | 36.1 | 28.0 | 33.3 | 38.0 | 33.4 | 18.7 | 7.2 | 11.8 | 23.2 | 18.5 |
| Female |  |  |  |  |  |  |  |  |  |  |
| By disability |  |  |  |  |  |  |  |  |  |  |
| People without disability |  |  |  |  |  |  |  |  |  |  |
| Total | 18,473,885 | 18,612,288 | 18,791,617 | 19,046,602 | 19,306,729 | 19,552,198 | 19,820,136 | 20,076,900 | 20,310,612 | 20,516,731 |
| Patients of complicated appendicitis | 4,595 | 4,689 | 4,925 | 5,071 | 5,172 | 2,722 | 839 | 679 | 2,215 | 2,014 |
| Incidence rate (crude) | 24.9 | 25.2 | 26.2 | 26.6 | 26.8 | 13.9 | 4.2 | 3.4 | 10.9 | 9.8 |
| Incidence rate (age-adjusted) | 24.4 | 24.7 | 25.7 | 26.2 | 26.3 | 13.2 | 3.6 | 2.9 | 8.4 | 7.5 |
| Severe (Grade 1-3) |  |  |  |  |  |  |  |  |  |  |
| Total | 362,377 | 384,746 | 394,354 | 393,771 | 388,670 | 384,014 | 381,117 | 379,366 | 380,395 | 382,051 |
| Patients of complicated appendicitis | 218 | 237 | 234 | 207 | 226 | 190 | 114 | 121 | 87 | 113 |
| Incidence rate (crude) | 60.2 | 61.6 | 59.3 | 52.6 | 58.1 | 49.5 | 29.9 | 31.9 | 22.9 | 29.6 |
| Incidence rate (age-adjusted) | 51.9 | 53.4 | 50.1 | 47.1 | 56.8 | 44.1 | 27.1 | 28.0 | 14.3 | 18.2 |
| Mild (Grade 4-6) |  |  |  |  |  |  |  |  |  |  |
| Total | 535,865 | 607,220 | 642,747 | 655,231 | 660,862 | 662,453 | 663,309 | 666,168 | 675,906 | 689,434 |
| Patients of complicated appendicitis | 212 | 235 | 261 | 276 | 276 | 151 | 69 | 60 | 221 | 177 |
| Incidence rate (crude) | 39.6 | 38.7 | 40.6 | 42.1 | 41.8 | 22.8 | 10.4 | 9.0 | 32.7 | 25.7 |
| Incidence rate (age-adjusted) | 31.7 | 27.0 | 37.8 | 35.9 | 34.8 | 21.5 | 7.3 | 7.3 | 11.8 | 8.8 |

**Supplementary Table 3. Incidence rate of complicated appendicitis according to type of disability**

| **Year** | **2008** | **2009** | **2010** | **2011** | **2012** | **2013** | **2014** | **2015** | **2016** | **2017** |
| --- | --- | --- | --- | --- | --- | --- | --- | --- | --- | --- |
| Male |  |  |  |  |  |  |  |  |  |  |
| By disability type |  |  |  |  |  |  |  |  |  |  |
| People without disability |  |  |  |  |  |  |  |  |  |  |
| Total | 17,672,422 | 17,827,464 | 18,040,675 | 18,319,643 | 18,595,507 | 18,845,674 | 19,143,089 | 19,427,929 | 19,662,987 | 19,857,936 |
| Patients of complicated appendicitis | 4,554 | 4,760 | 5,079 | 5,361 | 5,331 | 2,871 | 948 | 804 | 4,258 | 3,910 |
| Incidence rate (crude) | 25.8 | 26.7 | 28.2 | 29.3 | 28.7 | 15.2 | 5.0 | 4.1 | 21.7 | 19.7 |
| Incidence rate (age-adjusted) | 26.2 | 27.2 | 28.6 | 29.6 | 28.9 | 15.3 | 4.9 | 3.9 | 18.7 | 16.6 |
| Physical |  |  |  |  |  |  |  |  |  |  |
| Total | 760,428 | 792,620 | 806,337 | 806,216 | 799,436 | 791,137 | 782,994 | 777,019 | 770,072 | 762,351 |
| Patients of complicated appendicitis | 244 | 250 | 324 | 351 | 312 | 208 | 88 | 112 | 367 | 297 |
| Incidence rate (crude) | 32.1 | 31.5 | 40.2 | 43.5 | 39.0 | 26.3 | 11.2 | 14.4 | 47.7 | 39.0 |
| Incidence rate (age-adjusted) | 34.0 | 30.7 | 37.8 | 39.5 | 31.9 | 21.2 | 6.2 | 9.7 | 21.6 | 16.5 |
| Brain injury |  |  |  |  |  |  |  |  |  |  |
| Total | 128,078 | 137,573 | 140,687 | 141,274 | 141,097 | 139,385 | 137,754 | 138,431 | 138,824 | 139,313 |
| Patients of complicated appendicitis | 74 | 91 | 95 | 91 | 77 | 61 | 42 | 24 | 78 | 70 |
| Incidence rate (crude) | 57.8 | 66.1 | 67.5 | 64.4 | 54.6 | 43.8 | 30.5 | 17.3 | 56.2 | 50.2 |
| Incidence rate (age-adjusted) | 29.6 | 44.7 | 62.6 | 61.9 | 41.6 | 44.8 | 29.7 | 10.0 | 27.9 | 15.0 |
| Communication |  |  |  |  |  |  |  |  |  |  |
| Total | 277,581 | 294,289 | 304,726 | 307,618 | 307,004 | 305,217 | 303,463 | 303,401 | 313,263 | 327,307 |
| Patients of complicated appendicitis | 112 | 139 | 137 | 167 | 141 | 100 | 63 | 50 | 175 | 185 |
| Incidence rate (crude) | 40.3 | 47.2 | 45.0 | 54.3 | 45.9 | 32.8 | 20.8 | 16.5 | 55.9 | 56.5 |
| Incidence rate (age-adjusted) | 39.6 | 37.1 | 41.7 | 40.9 | 37.0 | 25.5 | 9.8 | 20.4 | 24.0 | 19.2 |
| Intellectual or psychological |  |  |  |  |  |  |  |  |  |  |
| Total | 108,594 | 115,879 | 120,794 | 125,107 | 128,641 | 132,429 | 136,447 | 141,005 | 145,317 | 149,235 |
| Patients of complicated appendicitis | 77 | 76 | 86 | 106 | 79 | 84 | 45 | 47 | 28 | 34 |
| Incidence rate (crude) | 70.9 | 65.6 | 71.2 | 84.7 | 61.4 | 63.4 | 33.0 | 33.3 | 19.3 | 22.8 |
| Incidence rate (age-adjusted) | 66.7 | 67.5 | 69.6 | 88.0 | 61.7 | 65.7 | 36.9 | 31.5 | 18.2 | 24.1 |
| Major internal organ |  |  |  |  |  |  |  |  |  |  |
| Total | 70,738 | 74,905 | 76,518 | 77,280 | 77,655 | 77,981 | 79,488 | 81,499 | 84,141 | 87,214 |
| Patients of complicated appendicitis | 69 | 78 | 88 | 83 | 73 | 74 | 72 | 70 | 98 | 110 |
| Incidence rate (crude) | 97.5 | 104.1 | 115.0 | 107.4 | 94.0 | 94.9 | 90.6 | 85.9 | 116.5 | 126.1 |
| Incidence rate (age-adjusted) | 115.9 | 95.4 | 125.5 | 108.1 | 103.6 | 87.2 | 62.5 | 97.5 | 111.1 | 94.8 |
| Female |  |  |  |  |  |  |  |  |  |  |
| By disability type |  |  |  |  |  |  |  |  |  |  |
| People without disability |  |  |  |  |  |  |  |  |  |  |
| Total | 18,473,885 | 18,612,288 | 18,791,617 | 19,046,602 | 19,306,729 | 19,552,198 | 19,820,136 | 20,076,900 | 20,310,612 | 20,516,731 |
| Patients of complicated appendicitis | 4,595 | 4,689 | 4,925 | 5,071 | 5,172 | 2,722 | 839 | 679 | 2,215 | 2,014 |
| Incidence rate (crude) | 24.9 | 25.2 | 26.2 | 26.6 | 26.8 | 13.9 | 4.2 | 3.4 | 10.9 | 9.8 |
| Incidence rate (age-adjusted) | 24.4 | 24.7 | 25.7 | 26.2 | 26.3 | 13.2 | 3.6 | 2.9 | 8.4 | 7.5 |
| Physical |  |  |  |  |  |  |  |  |  |  |
| Total | 487,930 | 547,780 | 572,052 | 575,034 | 572,479 | 568,222 | 563,933 | 560,953 | 556,581 | 551,826 |
| Patients of complicated appendicitis | 192 | 202 | 227 | 233 | 226 | 122 | 50 | 52 | 161 | 136 |
| Incidence rate (crude) | 39.4 | 36.9 | 39.7 | 40.5 | 39.5 | 21.5 | 8.9 | 9.3 | 28.9 | 24.6 |
| Incidence rate (age-adjusted) | 24.0 | 28.3 | 30.2 | 34.2 | 28.0 | 23.0 | 9.4 | 4.4 | 10.7 | 9.6 |
| Brain injury |  |  |  |  |  |  |  |  |  |  |
| Total | 93,619 | 101,601 | 104,899 | 105,391 | 105,600 | 104,581 | 103,796 | 104,340 | 104,590 | 104,823 |
| Patients of complicated appendicitis | 62 | 88 | 61 | 53 | 61 | 54 | 20 | 16 | 28 | 32 |
| Incidence rate (crude) | 66.2 | 86.6 | 58.2 | 50.3 | 57.8 | 51.6 | 19.3 | 15.3 | 26.8 | 30.5 |
| Incidence rate (age-adjusted) | 50.6 | 49.0 | 39.0 | 29.2 | 23.8 | 24.0 | 10.3 | 4.4 | 18.4 | 9.2 |
| Communication |  |  |  |  |  |  |  |  |  |  |
| Total | 191,442 | 208,447 | 219,716 | 223,581 | 224,169 | 223,338 | 222,687 | 222,559 | 232,567 | 247,810 |
| Patients of complicated appendicitis | 86 | 84 | 109 | 99 | 92 | 60 | 34 | 24 | 70 | 59 |
| Incidence rate (crude) | 44.9 | 40.3 | 49.6 | 44.3 | 41.0 | 26.9 | 15.3 | 10.8 | 30.1 | 23.8 |
| Incidence rate (age-adjusted) | 42.7 | 33.2 | 42.5 | 37.0 | 34.6 | 24.7 | 4.7 | 9.5 | 12.0 | 6.8 |
| Intellectual or psychological |  |  |  |  |  |  |  |  |  |  |
| Total | 82,087 | 88,257 | 92,209 | 95,437 | 97,569 | 100,017 | 102,772 | 105,850 | 108,974 | 111,725 |
| Patients of complicated appendicitis | 45 | 64 | 49 | 58 | 75 | 53 | 41 | 38 | 15 | 17 |
| Incidence rate (crude) | 54.8 | 72.5 | 53.1 | 60.8 | 76.9 | 53.0 | 39.9 | 35.9 | 13.8 | 15.2 |
| Incidence rate (age-adjusted) | 53.1 | 67.4 | 48.7 | 55.4 | 70.1 | 48.5 | 32.6 | 31.9 | 11.3 | 13.7 |
| Major internal organ |  |  |  |  |  |  |  |  |  |  |
| Total | 43,164 | 45,881 | 48,225 | 49,559 | 49,715 | 50,309 | 51,238 | 51,832 | 53,589 | 55,301 |
| Patients of complicated appendicitis | 45 | 34 | 49 | 40 | 48 | 52 | 38 | 51 | 34 | 46 |
| Incidence rate (crude) | 104.3 | 74.1 | 101.6 | 80.7 | 96.6 | 103.4 | 74.2 | 98.4 | 63.4 | 83.2 |
| Incidence rate (age-adjusted) | 136.4 | 60.4 | 111.6 | 66.2 | 149.6 | 98.2 | 68.3 | 102.8 | 32.4 | 50.4 |
